# 1-{4-[(1*H*-1,2,4-Triazol-1-yl)meth­yl]benz­yl}-1*H*-1,2,4-triazol-4-ium perchlorate

**DOI:** 10.1107/S1600536810002618

**Published:** 2010-02-06

**Authors:** Zhao-Jian Hu, Xiu-Kai Guo, Huan Xu, Ming-Yang He, Li Geng

**Affiliations:** a Key Laboratory of Fine Petrochemical Technology, Jiangsu Polytechnic University, Changzhou 213164, People’s Republic of China

## Abstract

In the crystal structure of the title compound, C_12_H_13_N_6_^+^·ClO_4_^−^, the cation, located about an inversion center, is monoprotonated, and one H atom is disordered over two sites on N atoms of the two triazole rings, each with an occupancy factor of 0.5. The perchlorate anion has *C*_2_ symmetry, the Cl atom and one O atom lying on the twofold rotation axis; the anion is thus disordered over two sites of equal occupancy. In the cation, the triazole ring makes a dihedral angle of 84.75 (7)° with the plane of the benzene ring. In the crystal, inter­molecular N—H⋯N hydrogen bonding between the triazole and triazolium rings links the cations into a wave-like supra­molecular chain. Weak inter­molecular C—H⋯N and C—H⋯O hydrogen bonding is also present.

## Related literature

For the versatile conformations of the flexible 1,4-bis­(1,2,4-triazol-1-yl-meth­yl)benzene ligand, see: Arion *et al.* (2003 ▶); Peng *et al.* (2004 ▶, 2006 ▶); Meng *et al.* (2004 ▶); Li *et al.* (2005 ▶); Ding *et al.* (2009 ▶).
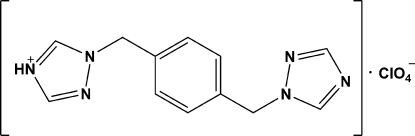


## Experimental

### 

#### Crystal data

C_12_H_13_N_6_^+^·ClO_4_^−^
*M* *_r_* = 340.73
Monoclinic, 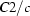

*a* = 15.140 (5) Å
*b* = 11.362 (3) Å
*c* = 10.408 (3) Å
β = 124.500 (5)°
*V* = 1475.5 (7) Å^3^
*Z* = 4
Mo *K*α radiation
μ = 0.29 mm^−1^
*T* = 297 K
0.20 × 0.15 × 0.14 mm

#### Data collection

Bruker APEXII CCD diffractometer
Absorption correction: multi-scan (*SADABS*; Sheldrick, 2003 ▶) *T*_min_ = 0.944, *T*_max_ = 0.961
4272 measured reflections
1436 independent reflections
1215 reflections with *I* > 2σ(*I*)
*R*_int_ = 0.021

#### Refinement

*R*[*F* ^2^ > 2σ(*F* ^2^)] = 0.054
*wR*(*F* ^2^) = 0.139
*S* = 1.09
1436 reflections
120 parameters
H-atom parameters constrained
Δρ_max_ = 0.28 e Å^−3^
Δρ_min_ = −0.61 e Å^−3^

### 

Data collection: *APEX2* (Bruker, 2007 ▶); cell refinement: *SAINT* (Bruker, 2007 ▶); data reduction: *SAINT*; program(s) used to solve structure: *SHELXS97* (Sheldrick, 2008 ▶); program(s) used to refine structure: *SHELXL97* (Sheldrick, 2008 ▶); molecular graphics: *DIAMOND* (Brandenburg, 2005 ▶); software used to prepare material for publication: *SHELXL97*.

## Supplementary Material

Crystal structure: contains datablocks I, global. DOI: 10.1107/S1600536810002618/xu2715sup1.cif

Structure factors: contains datablocks I. DOI: 10.1107/S1600536810002618/xu2715Isup2.hkl

Additional supplementary materials:  crystallographic information; 3D view; checkCIF report

## Figures and Tables

**Table 1:** Hydrogen-bond geometry (Å, °)

*D*—H⋯*A*	*D*—H	H⋯*A*	*D*⋯*A*	*D*—H⋯*A*
N3—H3*A*⋯N3^i^	0.86	1.86	2.690 (5)	162
C1—H1⋯O4^ii^	0.93	2.54	3.445 (12)	164
C3—H3⋯O2^iii^	0.93	2.55	3.403 (15)	152
C4—H4*A*⋯O4^iv^	0.97	2.46	3.430 (15)	176
C6—H6⋯N2^iv^	0.93	2.55	3.256 (4)	133

Symmetry codes: (i) 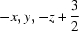; (ii) 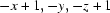; (iii) 
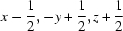
; (iv) 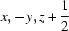.

**Fig. 1.**
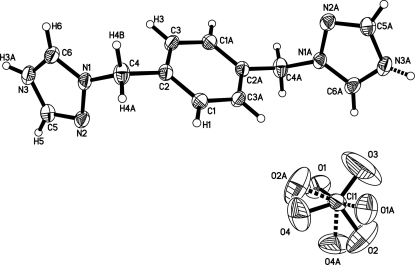
A view of the molecule of (I). Displacement ellipsoids are drawn at the 30% probability level. Symmetry code: (A) -x + 1/2, -y + 1/2, -z + 1.

**Fig. 2.**
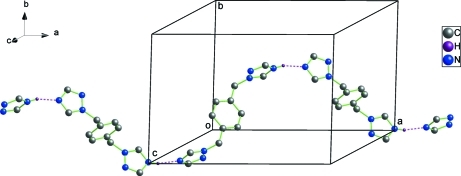
A one-dimensional hydrogen-bonding chain motif of (I).

## supplementary crystallographic information

### Comment

In recent years, there has been of great interest in the design and utilization of 1,2,4-triazole and its derivatives in coordination and biological chemistry for they represent the simple small molecular ligands. Among them, 1,4-Bis(1,2,4-triazol-1-yl-methyl)benzene (*L*) has attracted significant attention because of its versatile conformations arising from the flexible rotation of σ bonds of two methylene carbon atoms (C_sp3_) between the terminal triazole groups and the benzene ring (Arion, *et al.*, 2003; Peng, *et al.*, 2004, 2006; Meng, *et al.*, 2004; Li *et al.*, 2005; Ding, *et al.* 2009). To further understand the supramolecular behavior of this ligand, we report herein the crystal structure of the title compound, [C_12_H_13_N_6_]^+^.ClO_4_^-^(I).

A perspective view of (I), including the atomic numbering scheme, is shown in Figure 1. The monoprotonated cationic H*L*^+^ moiety of compound (I) crystallizes around an inversion center with a half molecule in the asymmetric unit. The anionic perchlorate is disorder over two positions related by a C_2_ axis, which crosses Cl1 and O3 atoms. Within each discrete *tran*-configurational cation, the triazole/triazolium ring makes a dihedral angle of 84.75 (7)° with the central benzene ring. Strong N—H···N interactions between triazole and triazolium groups join these cationic molecules into an infinite wavelike chain running along the crystallographic [1 0 1] direction (Figure 2). The final crystal structure results in a three-dimensional (3-D) hydrogen bonding network through the linkage of multiple C—H···N and C—H···O hydrogen bonds between the cationic subunits and perchlorate anions (Table 1).

### Experimental

Zn(ClO_4_)_2._6H_2_O (74 mg, 0.2 mmol) and 1,4-bis(1,2,4-triazol-1-yl-methyl)benzene (*L*) (48 mg, 0.2 mmol) was dissolved in a 8 ml ethanol-water mixture (V:V = 1:3) at room temperature. The colorless crystals were obtained after several days. Yield: 60% (based on *L*).

### Refinement

All hydrogen atoms on C atoms were positioned geometrically and refined as riding atoms, with C—H = 0.93 - 0.97 Å, and *U*_iso_(H)=1.2U_eq _(C). H atom on N atom of triazolium was firstly located in a difference Fourier map and then refined with restrained N—H = 0.86 Å. It is notable that triazolium hydrogen atom was assigned to half occupancy for charge balance.

### Crystal data

**Table:**

C_12_H_13_N_6_^+^·ClO_4_^−^	*F*(000) = 704
*M**_r_* = 340.73	*D*_x_ = 1.534 Mg m^−^^3^
Monoclinic, *C*2/*c*	Mo *K*α radiation, λ = 0.71073 Å
Hall symbol: -C 2yc	Cell parameters from 1942 reflections
*a* = 15.140 (5) Å	θ = 2.4–27.5°
*b* = 11.362 (3) Å	µ = 0.29 mm^−^^1^
*c* = 10.408 (3) Å	*T* = 297 K
β = 124.500 (5)°	Block, colorless
*V* = 1475.5 (7) Å^3^	0.20 × 0.15 × 0.14 mm
*Z* = 4	

### Data collection

**Table:**

Bruker APEXII CCD diffractometer	1436 independent reflections
Radiation source: fine-focus sealed tube	1215 reflections with *I* > 2σ(*I*)
graphite	*R*_int_ = 0.021
φ and ω scans	θ_max_ = 26.0°, θ_min_ = 2.4°
Absorption correction: multi-scan (*SADABS*; Sheldrick, 2003)	*h* = −12→18
*T*_min_ = 0.944, *T*_max_ = 0.961	*k* = −13→13
4272 measured reflections	*l* = −12→12

### Refinement

**Table:**

Refinement on *F*^2^	Primary atom site location: structure-invariant direct methods
Least-squares matrix: full	Secondary atom site location: difference Fourier map
*R*[*F*^2^ > 2σ(*F*^2^)] = 0.054	Hydrogen site location: inferred from neighbouring sites
*wR*(*F*^2^) = 0.139	H-atom parameters constrained
*S* = 1.09	*w* = 1/[σ^2^(*F*_o_^2^) + (0.0491*P*)^2^ + 2.663*P*] where *P* = (*F*_o_^2^ + 2*F*_c_^2^)/3
1436 reflections	(Δ/σ)_max_ < 0.001
120 parameters	Δρ_max_ = 0.28 e Å^−^^3^
0 restraints	Δρ_min_ = −0.61 e Å^−^^3^

### Special details

**Table:**

Geometry. All e.s.d.'s (except the e.s.d. in the dihedral angle between two l.s. planes) are estimated using the full covariance matrix. The cell e.s.d.'s are taken into account individually in the estimation of e.s.d.'s in distances, angles and torsion angles; correlations between e.s.d.'s in cell parameters are only used when they are defined by crystal symmetry. An approximate (isotropic) treatment of cell e.s.d.'s is used for estimating e.s.d.'s involving l.s. planes.
Refinement. Refinement of *F*^2^ against ALL reflections. The weighted *R*-factor *wR* and goodness of fit *S* are based on *F*^2^, conventional *R*-factors *R* are based on *F*, with *F* set to zero for negative *F*^2^. The threshold expression of *F*^2^ > σ(*F*^2^) is used only for calculating *R*-factors(gt) *etc*. and is not relevant to the choice of reflections for refinement. *R*-factors based on *F*^2^ are statistically about twice as large as those based on *F*, and *R*- factors based on ALL data will be even larger.

### Fractional atomic coordinates and isotropic or equivalent isotropic displacement parameters (Å^2^)

**Table:**

	*x*	*y*	*z*	*U*_iso_*/*U*_eq_	Occ. (<1)
C1	0.3304 (2)	0.1668 (2)	0.5558 (3)	0.0423 (6)	
H1	0.3848	0.1112	0.5929	0.051*	
C2	0.27311 (19)	0.1755 (2)	0.6225 (3)	0.0388 (5)	
C3	0.19301 (19)	0.2594 (2)	0.5662 (3)	0.0417 (6)	
H3	0.1545	0.2665	0.6106	0.050*	
C4	0.3004 (2)	0.0963 (3)	0.7575 (3)	0.0526 (7)	
H4A	0.3578	0.0433	0.7799	0.063*	
H4B	0.3260	0.1441	0.8494	0.063*	
C6	0.1572 (2)	0.0310 (2)	0.7933 (3)	0.0495 (6)	
H6	0.1735	0.0809	0.8748	0.059*	
C5	0.0862 (2)	−0.0993 (2)	0.6166 (3)	0.0471 (6)	
H5	0.0403	−0.1588	0.5514	0.057*	
N1	0.20807 (16)	0.02713 (17)	0.7242 (2)	0.0416 (5)	
N2	0.16400 (17)	−0.05709 (19)	0.6103 (2)	0.0466 (5)	
N3	0.07910 (18)	−0.0479 (2)	0.7274 (3)	0.0506 (6)	
H3A	0.0338	−0.0630	0.7501	0.061*	0.50
Cl1	0.5000	0.16865 (9)	0.2500	0.0749 (4)	
O1	0.3684 (4)	0.1843 (5)	0.1366 (6)	0.0902 (16)	0.50
O2	0.5109 (9)	0.1370 (12)	0.1316 (12)	0.136 (5)	0.50
O3	0.5000	0.2786 (5)	0.2500	0.254 (5)	
O4	0.5108 (10)	0.0799 (8)	0.3416 (13)	0.122 (3)	0.50

### Atomic displacement parameters (Å^2^)

**Table:**

	*U*^11^	*U*^22^	*U*^33^	*U*^12^	*U*^13^	*U*^23^
C1	0.0410 (12)	0.0427 (13)	0.0453 (13)	0.0003 (10)	0.0257 (11)	−0.0002 (10)
C2	0.0408 (12)	0.0412 (12)	0.0334 (11)	−0.0083 (10)	0.0203 (10)	−0.0022 (9)
C3	0.0451 (13)	0.0479 (13)	0.0432 (13)	−0.0057 (10)	0.0316 (11)	−0.0048 (10)
C4	0.0472 (14)	0.0639 (17)	0.0379 (13)	−0.0091 (13)	0.0188 (11)	0.0072 (12)
C6	0.0652 (17)	0.0506 (14)	0.0467 (14)	0.0094 (13)	0.0400 (13)	0.0098 (11)
C5	0.0482 (14)	0.0473 (14)	0.0451 (13)	−0.0006 (11)	0.0260 (12)	0.0074 (11)
N1	0.0500 (11)	0.0451 (11)	0.0342 (10)	−0.0005 (9)	0.0266 (9)	0.0069 (8)
N2	0.0533 (12)	0.0522 (12)	0.0382 (10)	−0.0024 (10)	0.0283 (10)	0.0015 (9)
N3	0.0535 (13)	0.0567 (13)	0.0569 (13)	0.0105 (11)	0.0405 (11)	0.0181 (11)
Cl1	0.1341 (12)	0.0393 (5)	0.0914 (9)	0.000	0.0878 (9)	0.000
O1	0.049 (2)	0.122 (4)	0.085 (3)	0.014 (3)	0.030 (3)	−0.006 (3)
O2	0.096 (6)	0.252 (15)	0.073 (4)	0.022 (8)	0.055 (4)	−0.004 (7)
O3	0.283 (10)	0.061 (3)	0.401 (14)	0.000	0.183 (10)	0.000
O4	0.129 (7)	0.105 (6)	0.120 (7)	0.012 (5)	0.064 (6)	0.061 (5)

### Geometric parameters (Å, °)

**Table:**

C1—C2	1.388 (3)	C5—N3	1.350 (3)
C1—C3^i^	1.389 (3)	C5—H5	0.9300
C1—H1	0.9300	N1—N2	1.367 (3)
C2—C3	1.385 (3)	N3—H3A	0.8600
C2—C4	1.515 (3)	Cl1—O3	1.249 (5)
C3—C1^i^	1.389 (3)	Cl1—O4^ii^	1.333 (8)
C3—H3	0.9300	Cl1—O4	1.333 (8)
C4—N1	1.464 (3)	Cl1—O2	1.381 (9)
C4—H4A	0.9700	Cl1—O2^ii^	1.381 (9)
C4—H4B	0.9700	Cl1—O1^ii^	1.653 (5)
C6—N1	1.319 (3)	Cl1—O1	1.653 (4)
C6—N3	1.324 (4)	O2—O4^ii^	0.843 (13)
C6—H6	0.9300	O4—O2^ii^	0.843 (13)
C5—N2	1.308 (3)	O4—O4^ii^	1.74 (2)
			
C2—C1—C3^i^	120.4 (2)	C6—N3—H3A	127.6
C2—C1—H1	119.8	C5—N3—H3A	127.6
C3^i^—C1—H1	119.8	O3—Cl1—O4^ii^	139.2 (5)
C3—C2—C1	118.9 (2)	O3—Cl1—O4	139.2 (5)
C3—C2—C4	121.0 (2)	O4^ii^—Cl1—O4	81.7 (10)
C1—C2—C4	120.1 (2)	O3—Cl1—O2	105.1 (5)
C2—C3—C1^i^	120.7 (2)	O4—Cl1—O2	114.4 (8)
C2—C3—H3	119.6	O3—Cl1—O2^ii^	105.1 (5)
C1^i^—C3—H3	119.6	O4^ii^—Cl1—O2^ii^	114.4 (8)
N1—C4—C2	112.1 (2)	O2—Cl1—O2^ii^	149.8 (11)
N1—C4—H4A	109.2	O3—Cl1—O1^ii^	83.8 (2)
C2—C4—H4A	109.2	O4^ii^—Cl1—O1^ii^	101.6 (6)
N1—C4—H4B	109.2	O4—Cl1—O1^ii^	87.8 (6)
C2—C4—H4B	109.2	O2—Cl1—O1^ii^	87.7 (5)
H4A—C4—H4B	107.9	O2^ii^—Cl1—O1^ii^	95.5 (5)
N1—C6—N3	108.7 (2)	O3—Cl1—O1	83.8 (2)
N1—C6—H6	125.6	O4^ii^—Cl1—O1	87.8 (6)
N3—C6—H6	125.6	O4—Cl1—O1	101.6 (6)
N2—C5—N3	113.2 (2)	O2—Cl1—O1	95.5 (5)
N2—C5—H5	123.4	O2^ii^—Cl1—O1	87.7 (5)
N3—C5—H5	123.4	O1^ii^—Cl1—O1	167.6 (4)
C6—N1—N2	110.3 (2)	O4^ii^—O2—Cl1	68.8 (9)
C6—N1—C4	128.9 (2)	O2^ii^—O4—Cl1	75.0 (11)
N2—N1—C4	120.8 (2)	O2^ii^—O4—O4^ii^	119.7 (14)
C5—N2—N1	103.0 (2)	Cl1—O4—O4^ii^	49.2 (5)
C6—N3—C5	104.8 (2)		
			
C3^i^—C1—C2—C3	−0.5 (4)	O3—Cl1—O2—O4^ii^	−163.6 (14)
C3^i^—C1—C2—C4	−178.9 (2)	O4—Cl1—O2—O4^ii^	27 (2)
C1—C2—C3—C1^i^	0.5 (4)	O2^ii^—Cl1—O2—O4^ii^	16.4 (14)
C4—C2—C3—C1^i^	178.8 (2)	O1^ii^—Cl1—O2—O4^ii^	113.4 (15)
C3—C2—C4—N1	58.5 (3)	O1—Cl1—O2—O4^ii^	−78.6 (15)
C1—C2—C4—N1	−123.1 (3)	O3—Cl1—O4—O2^ii^	25 (2)
N3—C6—N1—N2	−0.7 (3)	O4^ii^—Cl1—O4—O2^ii^	−155 (2)
N3—C6—N1—C4	−179.2 (2)	O2—Cl1—O4—O2^ii^	−171.0 (9)
C2—C4—N1—C6	−115.6 (3)	O1^ii^—Cl1—O4—O2^ii^	102.5 (15)
C2—C4—N1—N2	66.0 (3)	O1—Cl1—O4—O2^ii^	−69.4 (15)
N3—C5—N2—N1	−0.5 (3)	O3—Cl1—O4—O4^ii^	180.000 (3)
C6—N1—N2—C5	0.7 (3)	O2—Cl1—O4—O4^ii^	−15.6 (12)
C4—N1—N2—C5	179.3 (2)	O2^ii^—Cl1—O4—O4^ii^	155 (2)
N1—C6—N3—C5	0.3 (3)	O1^ii^—Cl1—O4—O4^ii^	−102.1 (6)
N2—C5—N3—C6	0.1 (3)	O1—Cl1—O4—O4^ii^	86.0 (6)

Symmetry codes: (i) −*x*+1/2, −*y*+1/2, −*z*+1; (ii) −*x*+1, *y*, −*z*+1/2.

### Hydrogen-bond geometry (Å, °)

**Table:**

*D*—H···*A*	*D*—H	H···*A*	*D*···*A*	*D*—H···*A*
N3—H3A···N3^iii^	0.86	1.86	2.690 (5)	162
C1—H1···O4^iv^	0.93	2.54	3.445 (12)	164
C3—H3···O2^v^	0.93	2.55	3.403 (15)	152
C4—H4A···O4^vi^	0.97	2.46	3.430 (15)	176
C6—H6···N2^vi^	0.93	2.55	3.256 (4)	133

Symmetry codes: (iii) −*x*, *y*, −*z*+3/2; (iv) −*x*+1, −*y*, −*z*+1; (v) *x*−1/2, −*y*+1/2, *z*+1/2; (vi) *x*, −*y*, *z*+1/2.
